# The NAC side of the fruit: tuning of fruit development and maturation

**DOI:** 10.1186/s12870-021-03029-y

**Published:** 2021-05-27

**Authors:** Sara Forlani, Chiara Mizzotti, Simona Masiero

**Affiliations:** Department of Biosciences, Università Degli Studi Di Milano, Via Celoria 26, 20133 Milan, Italy

**Keywords:** Fruit, NAC, Ripening, Senescence, Transcription factor

## Abstract

Fruits and seeds resulting from fertilization of flowers, represent an incredible evolutionary advantage in angiosperms and have seen them become a critical element in our food supply.

Many studies have been conducted to reveal how fruit matures while protecting growing seeds and ensuring their dispersal. As result, several transcription factors involved in fruit maturation and senescence have been isolated both in model and crop plants. These regulators modulate several cellular processes that occur during fruit ripening such as chlorophyll breakdown, tissue softening, carbohydrates and pigments accumulation.

The NAC superfamily of transcription factors is known to be involved in almost all these aspects of fruit development and maturation. In this review, we summarise the current knowledge regarding NACs that modulate fruit ripening in model species (*Arabidopsis thaliana* and *Solanum lycopersicum*) and in crops of commercial interest (*Oryza sativa*, *Malus domestica*, *Fragaria* genus, *Citrus sinensis* and *Musa acuminata*).

**Supplementary Information:**

The online version contains supplementary material available at 10.1186/s12870-021-03029-y.

## Background

It is widely accepted that fruits play a key role in the evolutionary history of angiosperms [1, 2]. Fruits protect seeds during their growth and development, as well as favour their dispersion, providing a remarkable evolutionary advantage [3]. At the same time, they are an essential nutritional component of our diet and one of the most important pillars of the world economy [4, 5]. Therefore, studies regarding the formation and ripening of fruit are crucial to unveil the mechanisms at play during fruit development, as this may lead to possible applications in the optimization of yield, quality, and post-harvest storage.

Transcription factors regulate the expression of many genes in different tissues and are classified according to their functional domains and binding specificity [6]. Among their many targets, they regulate the transcription of genes involved in environmental stress response [7, 8], response to pathogens [9], and fruit ripening [10, 11] all of which impact proper fruit development and maturation. Many transcription factors are known to be involved in such processes, as example, the MADS-box (MCM1/AGAMOUS/DEFICIENS/SRF) transcription factor family counts numerous key players of fruit development and maturation [12], together with the MYB (MYeloBlastosis) [13, 14] and the AP2/ERF (APETALA 2/Ethylene Response Factors) DNA binding proteins [15, 16]. Beside these wide groups of transcription factors, smaller families also participate to the regulation of fruit development and ripening like SPL (*SQUAMOSA* promoter binding protein-like), TCP (TEOSINTE BRANCHED 1/CYCLOIDEA/PROLIFERATING4 CELL FACTORS) and Dof (DNA binding with one finger) proteins [17–19]. However, one of the largest and most famous groups of plant-specific transcription factors known to be involved in such processes is the NAC (NAM/ATAF1/CUC2) superfamily [20–23]. In the next paragraphs, we discuss the pivotal role of NAC transcription factors in fruit development and maturation, focusing not only on fruit model species (*Arabidopsis thaliana* and *Solanum lycopersicum*) but also on other relevant species.

## Synopsis of NAC transcription factors

### A plant-specific family

NAC superfamily is a large group of plant-specific transcription factors whose name is an acronym of NAM, ATAF1,2, and CUC2, the first members characterized. NAM (No Apical Meristem) was characterized in *Petunia* x *hybrida*; in *nam* mutants, embryos fail to develop SAM (Shoot Apical Meristem), cotyledons are fused, and occasionally they display an abnormal number and distribution of organ primordia in flowers [24]. In *Arabidopsis thaliana*, *CUC2* (Cup-Shaped Cotyledon) displays high homology with the *NAM* sequence. CUC2 acts redundantly with CUC1, and the double mutant *cuc1 cuc2* fails to develop a SAM and its cotyledons are fused on both sides [25]. Arabidopsis Transcription Activator Factors (ATAF) are a subgroup of the NAC family which includes two of the first NAC proteins characterized, ATAF1 and 2, as being involved in stress response and senescence [26, 27]. For a deeper characterization of these transcription factors and their conserved structure, see below (paragraph 2.2 “NAC transcription factors’ structure” and 3.1 “Arabidopsis fruit and NAC transcription factors”).

Members of the NAC superfamily can be found in many different plant species (see Table S1). However, numbers and species are constantly increasing together with the improvement of genome annotations (see paragraph 3.3 “A constantly increasing number of species relies on NAC transcription factors”). In Table 1, we list the NAC transcription factors presented and discussed in the next paragraphs.

**Table 1:** NAC transcription factors described in this manuscript, listed in order of appearance

**Species**	**NAC TF**	**Gene ID/Genbank reference number**	**Main function in fruit**
*Arabidopsis thaliana*	*NARS1/ANAC056*/*NAC2*	AT3G15510	Embryogenesis and silique senescence
*Arabidopsis thaliana*	*NARS2/ANAC018*/*NAM*	AT1G52880	Embryogenesis and silique senescence
*Arabidopsis thaliana*	*AtNAP/ANAC029*	AT1G69490	Silique senescence
*Arabidopsis thaliana*	*ANAC058*	AT3G18400	Silique senescence
*Arabidopsis thaliana*	A*tJUB1/ANAC042*	AT2G43000	Silique growth
*Solanum lycopersicum*	*NOR*	Solyc10g006880	Ethylene biosynthesis, carotenoid accumulation, fruit softening
*Solanum lycopersicum*	*NOR-like1/SlNAC3 /SNAC4/SlNAC48*	Solyc07g063420	Seed development, carotenoid accumulation, ethylene biosynthesis, fruit softening
*Solanum lycopersicum*	*SlNAC1/SlNAC033*	Solyc04g009440	Fruit softening, pigmentation
*Solanum lycopersicum*	*SlORE1S02*	Solyc02g088180	Fruit senescence, sugar accumulation
*Solanum lycopersicum*	*SlNAP2/SNAC9/SlNAC19*	Solyc04g005610	Sugar and carotenoid accumulation, ethylene production
*Oryza sativa*	*OsNAC020*	Os01g01470	Grain size and weight
*Oryza sativa*	*OsNAC023*	Os02g12310	Grain size and weight
*Oryza sativa*	*OsNAC026*	Os01g29840	Grain size and weight
*Oryza sativa*	*ONAC127*	Os11g31340	Starch accumulation, carbohydrate transport
*Oryza sativa*	*ONAC129*	Os11g31380	Starch accumulation, carbohydrate transport
*Malus domestica*	*MdNAC1*	103,451,803	Ethylene signalling, ABA biosynthesis
*Malus domestica*	*MdNAC2*	103,446,449	Ethylene signalling
*Malus domestica*	*MdNAC47/MdNAC63*	MG099900	Ethylene production
*Malus domestica*	*NAC18.1*	103,436,128	Fruit softening, harvest date
*Malus domestica*	*MdNAC52*	MG099889	Anthocyanin accumulation
*Malus domestica*	*MdNAC042*	818,902	Anthocyanin accumulation
*Fragaria x ananassa*	*FaNAC006*	FvH4_1g27900	Fruit softening
*Fragaria x ananassa*	*FaNAC021*	FvH4_3g04630	Fruit softening
*Fragaria x ananassa*	*FaNAC022*	FvH4_3g08490	Fruit softening
*Fragaria x ananassa*	*FaNAC035*	FvH4_3g20700	Fruit softening, pigment and sugar accumulation
*Fragaria x ananassa*	*FaNAC042*	FvH4_4g23130	Fruit softening
*Fragaria x ananassa*	*FaNAC092*	FvH4_6g48120	Fruit softening
*Fragaria chiloensis*	*FcNAC1*	KP966107	Cell wall remodelling
*Citrus sinensis*	*CitNAC62*	Ciclev10019368m	Lowering of citric acid content
*Citrus sinensis*	CsNAC	EF596736	Response to citrus peel pitting
*Musa acuminata*	*MaNAC1/ MaNAC087*	Achr6T27000^a^	Ethylene signalling
*Musa acuminata*	*MaNAC2/ MaNAC092*	Achr6T31585^a^	Ethylene signalling
*Musa acuminata*	*MaNAC3*	Achr9T27530^a^	Ethylene signalling
*Musa acuminata*	*MaNAC4*	Achr7T00860^a^	Ethylene signalling
*Musa acuminata*	*MaNAC5/ MaNAC140*	Achr9T26140^a^	Ethylene signalling, response to biotic stress
*Musa acuminata*	*MaNAC6*	Achr11T00880^a^	Ethylene signalling

^a^ID as reported in [28]

**Fig. 1:**
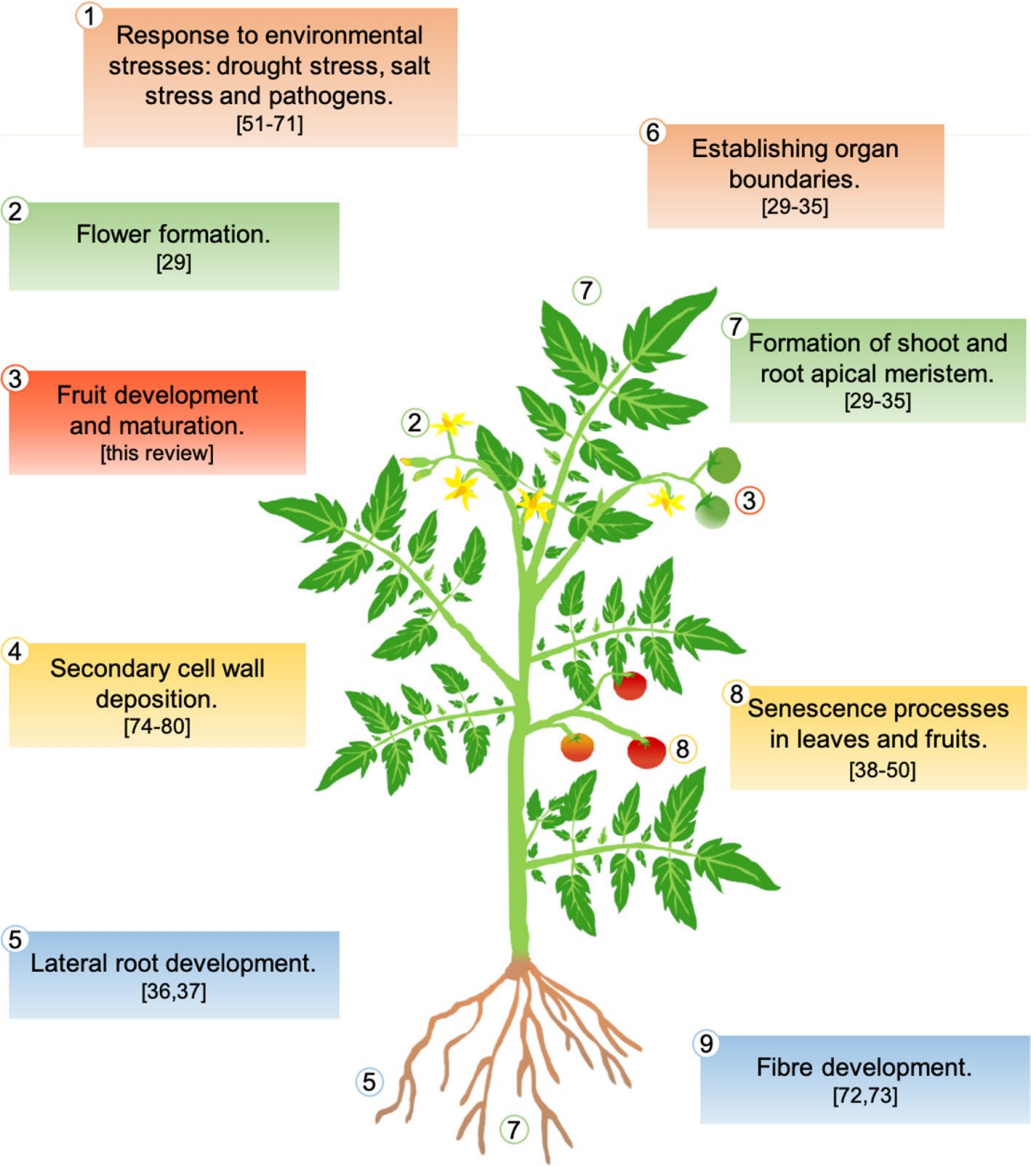
NACs play a role in numerous processes during a plant’s lifecycle, summarized in the figure using *Solanum lycopersicum* as representative plant. Each box represents a process, significant examples of NAC TFs involved are reported as references between brackets

NAC family is one of the largest groups of transcription factors and they modulate several processes during a plant’s lifetime (Fig. 1). They participate in various developmental programmes, such as the formation of shoot and root apical meristem during embryogenesis, interacting with homeotic proteins and establishing organ boundaries [29–35]. Some NAC transcription factors have also been shown to play a role in lateral root development [36, 37] and in flower formation [29]. They also regulate senescence processes in leaves and fruits [38–50].

NAC transcription factors mainly modulate the response to environmental stresses [51–58], such as drought stress [59–65] and salt stress [66, 67]. At the same time, NAC transcription factors also regulate the stress response triggered by pathogens [52, 55, 68–71]. Furthermore, they participate in fibre development [72, 73], and secondary cell wall deposition [74–79] through the binding of a NAC-specific sequence in the promoter of the target genes [80]. Among several other targets, NACs regulate MYB transcription factors involved in secondary cell wall and lignin biosynthesis. Further still, they target either, other NACs or enzymes involved in cell wall modification and programmed cell death [80, 81]. As important regulators of plant processes, NAC transcription factors respond to phytohormones [82] such as abscisic acid (ABA) [83], ethylene [84], cytokinins [85], jasmonic acid [86], gibberellins [87, 88], and auxin [36, 89].

Among all the regulatory processes they govern, NAC transcription factors also modulate fruit development and maturation. Hereby, a complete overview of the fruit-related NACs, identified and characterized so far, will be provided.

### NAC transcription factors’ structure

**Fig. 2:**
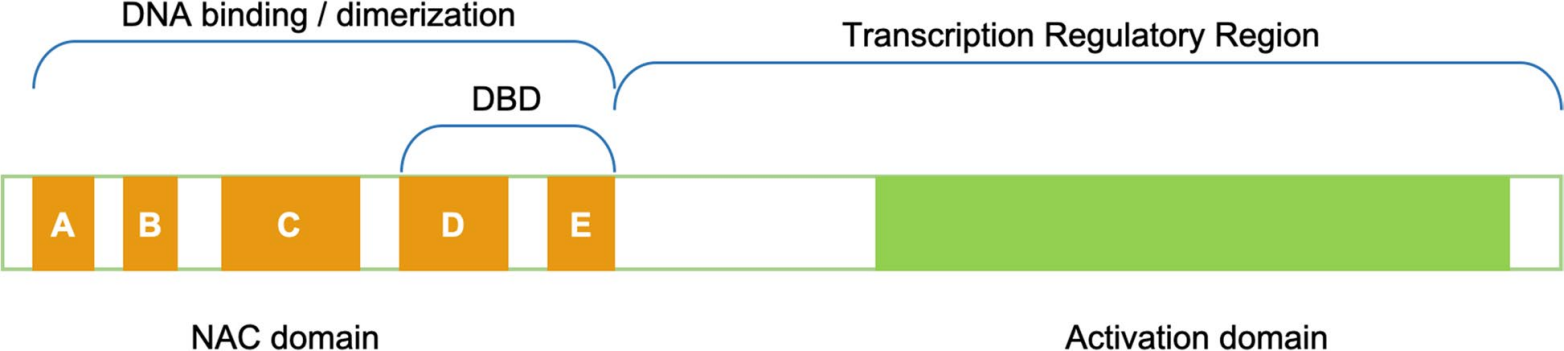
Structural domains and subdomains of NAC TFs

The modular structure of NAC TFs is characterized by a highly conserved N-terminal NAC domain (of about 150 amino acids) and a C-terminal Transcription Regulatory Region (TRR) which is more divergent (Fig. 2) [90, 91]. The NAC domain is responsible for the DNA binding activity and it is divided into five subdomains A-E [92, 93]. The subdomain A is involved in the formation of functional dimers, while the C and D subdomains, which are highly conserved, contain several positively charged amino acids necessary to interact with the DNA. The B and E subdomains are divergent, and they might be responsible for the functional diversity of NAC genes [90, 94]. Putative Nuclear Localization Signals (NLS) have been detected in C and D subdomains, while the DNA Binding Domain (DBD) is a 60 amino acid region located within subdomains D and E [92, 93]. In many NAC proteins, the NLS is present at the N-terminal end, however, Mohanta and collaborators also described some NLS placed in the C-terminal region [23]. They also described NAC TFs that contain more than one NLS, as many as four NLS in a single NAC protein. The NLS is characterized by the presence of a cluster of positively charged amino acids, and NAC proteins can contain single or more than one cluster defining mono-, bi-, or multi-partite motifs [23].

The N-terminal DBD is also involved in the formation of homo- and/or hetero-dimers: NAC TFs can bind DNA as homo- or hetero-dimers and the dimerization is necessary for a DNA stable binding [20, 95]. In soybean, it has been demonstrated that the D subdomain contains a hydrophobic Negative Regulatory Domain (NRD) to suppress the transcriptional activity [96]. Such a transcriptional repressor motif was recently identified in several plant species [23].

At the C-terminal, the presence of several group-specific motifs in the TRR allow the activation or repression of the transcription [23]. Its divergence determines the function of each NAC protein and modulates the interaction between NAC TFs and their target proteins. Welner and collaborators [95] suggested that NAC genes present a conserved consensus sequence for specific DNA recognition that increases DNA binding affinity. Recently, this consensus sequence was detected in 160 plant species [23].

Several NACs possess a transmembrane domain; it can be located at both the terminal ends of the proteins but were prominently found at the C-terminal end [23].

In 2004, Ernst and collaborators solved the NAC domain’s structure of ANAC019 by X-ray crystallography [90]. Some years later, Chen and collaborators determined the crystal structure of the NAC domain of the STRESS-RESPONSIVE NAC1 (SNAC1) protein in rice [94]. The NAC domain consists of a twisted antiparallel β-sheet, used for DNA binding, packed between an N-terminal α-helix on one side and a short helix on the other [90]. Several amino acids located in subdomains C, D and E were identified as having biochemical functions crucial for DNA binding [90, 91, 94].

The sequence of the NAC binding site has been addressed with several techniques (X-ray, SELEX, EMSA, footprint, PBM, ChIP). The binding site contains the CGT[AG] core motif, and the flanking sequences are involved in the determination of the target promoter binding affinity and specificity (for a review see [97]).

## Same family, different species

The NAC superfamily includes only plant-specific transcription factors, widespread in an increasing number of species. First, we will focus on model species, such as *Arabidopsis thaliana* and *Solanum lycopersicum*, for their role as reference plants in studies focused on fruit development, ripening and senescence [98].

### *Arabidopsis thaliana* siliques and NAC transcription factors

The first NACs described as regulators of fruit senescence were *NARS1* and *NARS2* (*NAC-REGULATED SEED MORPHOLOGY1* and *-2*, also known as *ANAC056*/*NAC2* and *ANAC018*/*NAM*, respectively). NARS1 and NARS2 regulate embryogenesis, through the control of ovule integument development and degeneration, seed morphogenesis, and silique senescence [99]. While the single mutants do not show any phenotype, the double mutant *nars1 nars2* has delayed silique senescence. Currently, no molecular data are available regarding NARS1 and/or NARS2’s involvement in hormonal signal pathways. It has been shown that NARS1 and/or NARS2 work to ease an intracellular environment triggered by programmed cell death in the integument, and similarly could also occur in the silique. Recently, Ma and colleagues [50] proposed *NARS1* and *NARS2* as *NOR* (*NONRIPENING*) homologs. NOR is a NAC transcription factor involved in tomato fruit ripening (see below, [100]). However, on the basis of their expression pattern and their phenotype, Ma and co-workers [50] concluded that NARS1 and NARS2 act differently from NOR. Indeed, while *NARS1* and *NARS2* are expressed in aging leaves, as well as in ripening fruits, in *nars1 nars2* mutant senescence was delayed only in siliques, while no effect was observed in leaves. This suggests a different mode of action compared to NOR.

*AtNAP* (*NAC-LIKE, ACTIVATED BY AP3/PI*, *ANAC029*) was initially described as promoter of leaf senescence [38], but was later shown to promote silique maturation as well [43]. Indeed, *AtNAP* messenger accumulates with fruit maturation progression and *atnap* siliques delay senescence by 4–5 days. Moreover, the ethylene and respiratory surges are decoupled, and exogenous ethylene treatments cannot anticipate the respiratory surge [43]. Few genes, whose products participate in ethylene biosynthesis, perception, and signal transduction pathways, were downregulated in *atnap* mutant. However, it is still not clear how the changes in the expression of these genes could affect the senescence in mutant plants. AtNAP might be NOR orthologue, but such conclusion is not fully supported [43].

The role of few other Arabidopsis NAC proteins has been described in siliques, among them NAC058 which represses silique senescence. In the *nac058* knock-down mutant the maturation of the fruit is precocious as demonstrated by the premature yellowing, the reduced chlorophyll content and the reduced photosynthetic performance [101].

*JUNGBRUNNEN1* (*JUB1/ ANAC042*) is not involved in the maturation process rather in the growth of the silique. The silique of plants that overexpress *JUB1* are shorter than normal, but it is interesting to report that in tomato the overexpression of *AtJUB1* delays fruit ripening by 6 days [87, 88]. The overexpression of *AtJUB1* in tomato represses several ethylene-related ripening genes such as *ACS* (ACC synthase) and *ACO* (ACC oxidase, [88]). In Arabidopsis and in tomato, AtJUB1 represses the transcription of *GA3ox1*(*GA 3-oxidase1*) and *DWF4* (*DWARF4*), genes important for gibberellin (GA) and brassinosteroid (BR) biosynthesis, thus phenocopying GA and BR deficiency. AtJUB1 also directly represses *PIF4* (*PHYTOCHROME INTERACTING FACTOR4*), a positive regulator of cell elongation, and activates the *DELLA* genes, repressors of cell proliferation and expansion [87, 88].

### Tomato fruit and their NAC transcription factors

The complete sequencing of the tomato genome [102] provided a fundamental tool for the prediction and identification of numerous genes. 104 *SlNAC* genes have been identified so far [103], mapping to all 12 chromosomes.

The *nor* mutant fails to produce the climacteric peak of ethylene, thereby causing an arrested ripening [100, 104] which cannot be rescued by exogenous application of ethylene [105, 106]. Positional cloning demonstrated that *NOR* encodes a NAC transcription factor [105, 106]. Another spontaneous allele of *NOR* is *alcobaca* (*alc*) found in the Penjar tomatoes which have a very long shelf life [107, 108]. The *alc* allele is weaker compared to *nor*, but its ripening delay is comparable to the newer *nor* alleles that have been recently obtained using the CRISPR-Cas9 methodology [108–111]. All these alleles are caused by amorphic mutations, and mutant plants display milder phenotypes compared to the classical *nor* mutant, that is actually a gain-of-function [111–113]. Similar results have also been obtained with CRISPR *null* alleles of other tomato transcription factors involved in fruit maturation, such as *RIN* and *CNR* [111]. *RIN* encodes a MADS-box transcription factor [114, 115] that directly binds *NOR* promoter [116–118]*.* However, Wang and collaborators [111] have recently suggested that such transcription factors might act redundantly in a complex network that integrates multiple signals, more so than being master regulators alone. Indeed, their effect on ethylene production, lycopene accumulation and other ripening traits has been shown to be quantitative and additive [119]. Moreover, although both *NOR* and *RIN* play a role in fruit senescence, only *NOR* has been found involved in the mediation of pathogen susceptibility in ripening fruit [120].

Some NAC transcription factors, able to influence fruit development and maturation, were initially classified as stress-responsive genes, and for this reason *NAC* genes are often referred to using different names, complicating literature data screenings [121]. *NOR-like1* was originally identified as *SlNAC3*, a negative regulator of drought stress under the control of ABA [122]. Later, analysing RNA interference lines, *SlNAC3* was revealed to be essential for appropriate embryo and endosperm development in seeds [123]. *Polygalacturonase-2 (PG-2)* was eventually isolated as one of SlNAC3’s direct targets through co-immunoprecipitation assays [124]. At the same time, Zhu and collaborators also referred to *NOR-like1* as *SNAC4* (*SlNAC48*), identifying it as regulator of salt stress and drought tolerance [125]. It shares 49.2% of nucleotide sequence with *NOR* and its disruption causes a decrease in carotenoid accumulation, chlorophyll breakdown and ethylene biosynthesis [126]. Moreover, *NOR-like1* can bind directly both *RIN* and *NOR*, suggesting that it could act upstream of these ripening regulators, and its predicted tertiary structure reveals a high degree of similarity with ANAC072, involved in chlorophyll degradation during leaf senescence [126–128]. Recently, *NOR-like1* has been shown to directly bind the promoter of genes involved in ethylene production, fruit firmness and colour change [129].

Tweneboah and Oh [55] summarized the stress-related NAC transcription factors in different Solanaceae and, as pointed out previously, some of them participate in fruit development and ripening. As an example, SlNAC1 (also known as SlNAC033) participates in heat and chilling tolerance [130, 131] and defence against *Pseudomonas syringae* [132], thus modulating biotic and abiotic stress responses. SlNAC1 binds the regulatory regions of genes related to ethylene or lycopene biosynthesis [133] and its suppression or overexpression can alter fruit softening and pigmentation [133, 134].

*SlORE1S02*, *SlORE1S03*, and *SlORE1S06* are the orthologues of the Arabidopsis *ORESARA1* (*ORE1*/*NAC092*) [135]. ORESARA means “long-living” in Korean; indeed, the *ore1* mutant delays leaf senescence in Arabidopsis [136]. *SlORE1S02*, *SlORE1S03*, *SlORE1S06* and *ORE1* are all regulated by the microRNA *miR164*. In tomato, they are expressed in leaves where their overexpression accelerates senescence. The reduction of *SlORE1S02* via RNAi interference leads to increased carbon assimilation, consequently transgenic plants show a higher harvest index with no consequences on fruit size. Transgenic fruit delay senescence and also accumulate more soluble solids in ripe fruit [135].

*SlNAP2* impacts fruit yield and metabolism, since its inhibition causes fruits to retain more sugars [83]. This transcription factor is structurally similar to *AtNAP* (described in the previous paragraph [43]). Kou and co-workers [127] described *SlNAP2* naming it *SNAC9* (*SlNAC19*). Fruit of *slnac19* exhibit a reduced carotenoid content and ethylene production [137]. The same phenotype has been observed in *nor-like1* fruits, but they accumulate more ABA than *SlNAC19* ones [137]. *SlNAP2* modulates *NOR* expression [50] and, together with *NOR* and *NOR-like1*, it is considered one of the key tomato ripening regulators [138].

## A constantly increasing number of species relies on NAC transcription factors

Beside model species for dry and fleshy fruits, NAC transcription factors have been identified and analysed in many other species of commercial interest. Here are listed the most recent findings in a few select species. We will review their role in *Oryza sativa*, *Malus domestica*, *Fragaria* genus, *Citrus sinensis* and *Musa acuminata*.

### Rice (*Oryza sativa*)

Although grains do not resemble the classic idea of fruit, they are actually composed of fruit coat strongly bound to the seed coat, and they enclose the germ and the endosperm. This non-canonical one-seeded fruit is produced by cereals, such as rice, barley and wheat, and it is called caryopsis.

One of the most widely cultivated cereals is rice, and for this reason it represents the topic of many researches. In rice, NAC transcription factors are involved in biotic and abiotic stress [52, 139]. *OsNAC020*, *OsNAC023* and *OsNAC026* have been identified as grain-specific conserved NAC genes in rice [140]. Indeed, previous studies on *OsNAC020*, *OsNAC023* and *OsNAC026* detected a higher expression in caryopsis [141]. Moreover, OsNAC020 and OsNAC023 can dimerize with OsNAC026 and localize in the nucleus to regulate genes involved in seed size and weight [142].

Since rice seeds are mostly intended for human consumption, their composition is one of the principle features to be considered. *ONAC127* and *ONAC129* are expressed in caryopsis and the corresponding gene products regulate starch accumulation and genes related to carbohydrates transport during grain filling [143].

Another important commercial feature is the grain yield, determined by numerous factors, e.g. resistance to drought stress and shoot branching. Overexpression of *OsNAC5*, *OsNAC9* and *OsNAC10* causes changes in root diameter and architecture leading to increased drought tolerance and grain yield [144–146]. Similarly, *OsNAP* and *SNAC1* overexpression leads to a greater seed production in restricted water conditions [147, 148]. However, it is interesting to note that not all the NAC transcription factors involved in drought stress mitigation can increase the grain yield, as for *ONAC022* [149]. Shoot branching can eventually influence grain yield. For example, *OsNAC2* regulates shoot branching [150] and plant height [151]. *OsNAC2* is negatively regulated by *miR164b*; if the sequence is mutated to be resistant to *miR164b*, the final grain number increases [152].

### Apple (*Malus domestica*)

In the apple cultivar “Golden delicious”, Wang and Xu [153] found that MdNAC1 and MdNAC2 interact and are co-expressed, respectively, with the ethylene receptors *MdRTE1a* (*Malus domestica REVERSION-TO-ETHYLENE SENSITIVITY1a*) and *MdRTE1b*. This pinpoint a possible involvement in pome growth and ripening. Later, *MdNAC1* was also found to be responsible for plant height and drought tolerance, since its overexpression leads to a dwarf phenotype [154] with reduced water loss and a stable photosynthetic rate [155]. Moreover, Jia and co-workers hypothesized that *MdNAC1* controls the biosynthesis of ABA [154].

NAC transcription factors mediate ethylene and auxin crosstalk in apple, especially when the production of ethylene is impaired [156], as already suggested in Arabidopsis [91].

Zhang and collaborators [157] found that 13 NAC genes are differentially expressed in numerous tissues during fruit growth and ripening. The evaluation of their response to 1-MCP treatment and ethylene exposure indicates that NACs could regulate pome development in both an ethylene-dependent and independent manner. The mediation of ethylene signalling was further confirmed by MdNAC47, which directly binds the positive regulator of ethylene biosynthesis *MdERF3* (*Malus domestica ETHYLENE RESPONSE FACTOR*), modulating salt stress tolerance [158]. MdNAC47 was so called since it was thought to be the orthologue of Arabidopsis *ANAC047*, however its genomic locus has been recently annotated as *MdNAC63* [159].

Fruit firmness, an important trait in post-harvest conservation, is also controlled by NAC transcription factors. In particular, the NAC18.1 protein displays high similarity with tomato NOR, and could be a major determinant of fruit softening and harvest date [160, 161]. Another process typical of the ripening of fleshy fruits is the accumulation of flavonoids such as anthocyanins, which give the fruit colour to attract frugivorous animals [162]. *MdNAC52* overexpression leads to anthocyanin accumulation in apple calli and it can induce the expression of *MdMYB9* and *MdMYB11*, regulating proanthocyanidin biosynthesis [163]. Recently, MdNAC042 was also discovered to positively correlate with anthocyanin content in red apples, regulating pigmentation through dimerization with MdMYB10 [164].

### Strawberry (*Fragaria genus*)

112 NAC genes have been identified in the commercial strawberry (*Fragaria x ananassa*), thanks to the comparison with the woodland strawberry (*Fragaria vesca*) genome [165]. Six of them are associated with fruit ripening and senescence: *FaNAC006*, *FaNAC021*, *FaNAC022*, *FaNAC035*, *FaNAC042*, *FaNAC092*. Their expression pattern correlates with anthocyanin biosynthesis [166] and their products play a role in tissue softening [167]. Recently, FaNAC035 was demonstrated to regulate ripening by controlling fruit softening and pigment and sugar accumulation. Moreover, it regulates ABA biosynthesis and signalling and cell wall degradation and modification [168].

*FaNAC087* and *FaNAC038* are negatively modulated by *miRNA164* [169], as happens in *Arabidopsis thaliana* with *ORE1* [170]. Since *FaNAC087* and *FaNAC038* increase their expression in the last stages of ripening [169], Li and co-workers [171] analysed their regulation in post-harvest storage conditions, confirming the negative correlation between *miRNA164* and its NAC messenger targets. The role of sRNAs as regulators of post-harvest shelf life has been recently corroborated, highlighting that NAC and other families of transcription factors represent important targets [172].

The commercial strawberry (*Fragaria x ananassa*) was obtained by crossing the parental species *Fragaria chiloensis* and *Fragaria virginiana*. Due to great interest in its limited post-harvest period, transcription factors involved in fruit ripening regulation and cell wall remodelling have been characterized in *Fragaria chiloensis*, including NAC TFs. Among them, FcNAC1 which interacts with FcPL (*Fragaria chiloensis* pectin lyase), contributing to cell wall remodelling [173].

### Orange (*Citrus sinensis*)

Given the economic and nutritional importance of sweet orange, the discovery of the regulators of fruit quality and duration have always been an essential goal to reach. In orange, transcription factors belonging to the NAC superfamily represent one of the key elements of these processes. NAC genes differentially expressed between a late-ripening mutant and a wild type variety of sweet orange have been identified [174], among them *NAC61*, *NAC74*, *NAC84* and *RD26* (*RESPONSIVE TO DESICCATION 26*). In particular, *RD26* displays high correlation with fructose and glucose accumulation; the Arabidopsis orthologue *RD26*/*ANAC072* is a transductor of the ABA signal [175] and activates the expression of genes whose products participates in chloroplast protein degradation during leaf senescence [176].

*CitNAC* is expressed in peel and pulp during orange fruit ripening and is phylogenetically similar to *AtNAP* [177]. This suggests the possible involvement of *CitNAC* in sweet orange fruit development and senescence.

In order to lower the citric acid content in mature fruit, CitNAC62 acts in a synergic way with CitWRKY1 modulating *CitAco3*, an aconitate hydratase involved in the catabolism of citric acid [178].

As in tomato, stress-related NAC transcription factors play an active role in determining fruit quality, thus influencing the postharvest conservation. Fan and co-workers [179] point out that CsNAC, the orthologue of Arabidopsis ATAF1, participates to citrus peel pitting, a disorder that affects the quality and the economic value of citrus fruits. NAC transcription factors are also involved in the response to cold storage, probably increasing the anthocyanin content [180]. Mitalo and collaborators [181] have found that the NAC superfamily participates in transcriptional cascades whose products help to counteract citrus greening, a destructive disease for citrus fruits (Bove, 2006). They may also be involved in a similar resistance process in lemon fruit [181]. In particular, *NAC-1* and *RD26* transcripts are detected during the symptomatic and asymptomatic phases of citrus greening [182]. In addition, many other citrus NAC transcription factors involved in response to multiple stresses have been found [183]. However, further studies are required to unveil their role in the determination of fruit quality and proper development.

### Banana (*Musa acuminata*)

Studies on NAC superfamily in banana started with the prediction of loci potentially coding for such transcription factors, and the definition of orthologous groups comparing sequences from monocots and dicots [28]. Recent works implemented the previous findings, identifying 181 NACs mapped in all the 12 chromosomes [184]. 10 *MaNACs* were associated with ripening in ethylene-treated banana fruits, some of them carrying ethylene responsive elements in their promoter. Six of them (*MaNAC1* to *MaNAC6*) have already been previously characterized as part of the ethylene mediated fruit ripening [185]. *MaNAC1* (*MaNAC087*) is known for its role in both stress tolerance and fruit maturation, it is induced after cold stress and physically interacts with cold signalling pathway elements [185]. Moreover, MaNAC1 interacts with MaEIL5 (*Musa acuminata* ETHYLENE INSENSITIVE 3-like 5), a downstream component of the ethylene signalling pathway [186]. MaNAC2 (MaNAC092), like MaNAC1, directly interacts with MaEIL5 and its expression is upregulated by ethylene both in the peel and pulp of banana fruit [186]. Additionally, the expression of *MaNAC1* and *MaNAC2*, together with *MaNAC5* (*MaNAC140*), is upregulated in fruit after infection with *Colletotrichum musae* [187]. Furthermore, MaNAC5 can interact with WRKY transcription factors and activate pathogenesis-related genes to counteract the disease [187]. Other NAC transcription factors characterized so far in banana play a role in leaves senescence [188] or drought stress [189, 190]. Further studies are necessary to deepen the role of NAC superfamily in banana fruit ripening.

## Conclusions

**Fig. 3:**
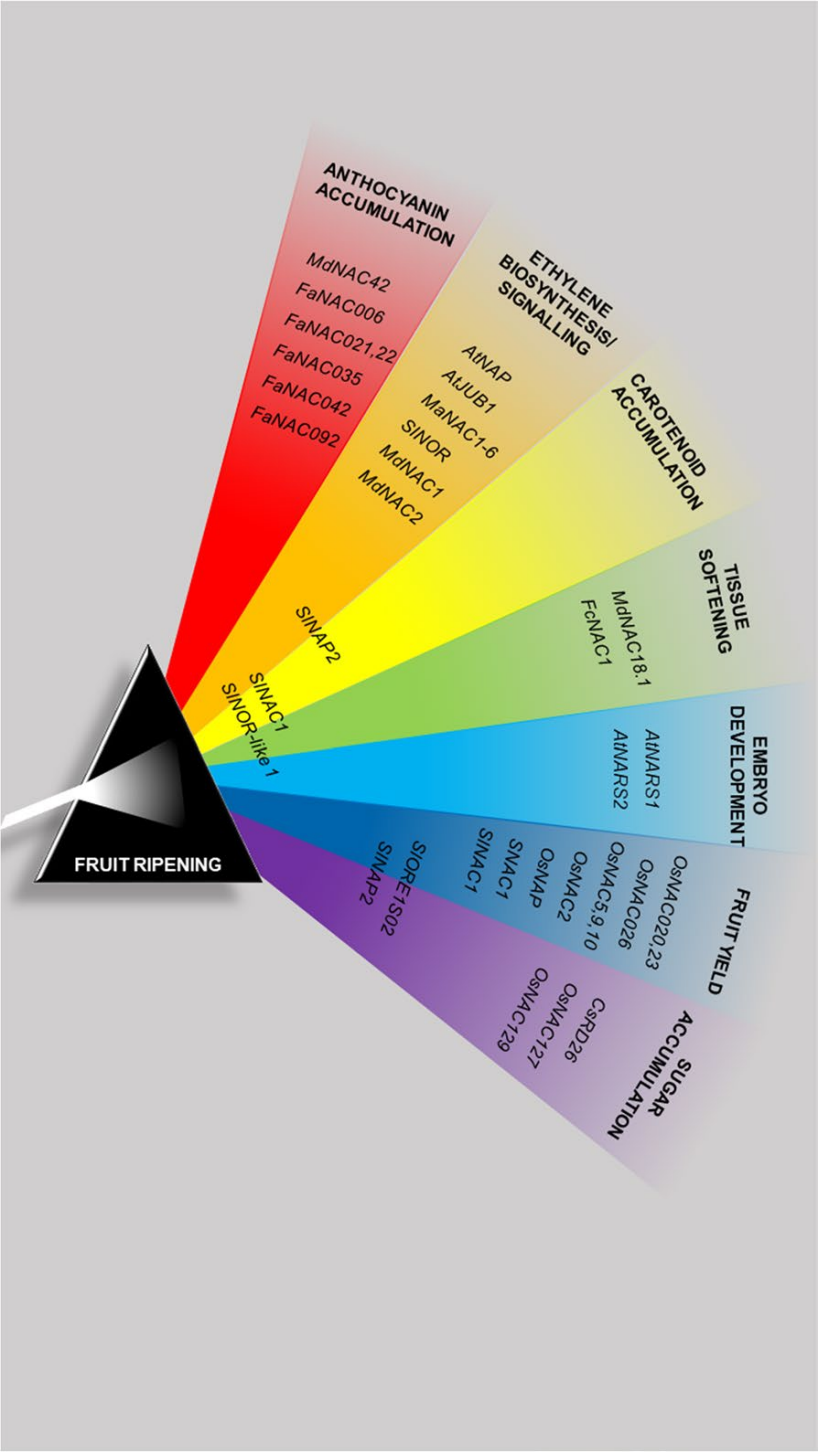
Overview of the principle fruit-related processes which NAC transcription factors are involved in. Each colour represents a specific aspect of fruit ripening, and the NAC genes listed in this review are located accordingly

The economic and dietary relevance of fruit is an important stimulus to explore fruit maturation and senescence in non-model species. In respect to fruit maturation, a pivotal role is played by NAC transcription factors and therefore they are proposed as interesting targets to modulate development and ripening, and to prolong fruit shelf life. In this work we provide a synopsis of the fruit-related NAC transcription factors identified in model species and in major crop species (Fig. 3). The processes of maturation and ripening must be fine-tuned to ensure the protection of growing seeds and the correct release of offspring. For this reason, they are regulated by a network of interconnected transcription factors belonging to different families.

Unfortunately, the non-model species have yet to be deeply investigated but, from the data available, it is clear that NAC transcription factors represent a conserved family necessary for the regulation of fruit development, maturation and post-harvest life. Due to their ubiquitous presence in numerous species, unveiling NAC TF regulation network could represent a potential tool for the regulation of post-harvest fruit conservation and pathogen resistance in economically relevant species. Within this view, as suggested by Singh and collaborators [191], future researches could focus on the analysis of redundancy among these TFs, in order to identify pivotal players in the regulation of fruit maturation.

In parallel, the identification of NAC interactors could reveal potential inhibitors or enhancers of their activity, helping the conservation and response to pathogens in commercial fruits.

## Supplementary Information

**Additional file 1: Table S1.** Number of NAC transcription factors identified in several plant species.

## Data Availability

Not applicable.

## Abbreviations

ABA: Abscisic acid
DBD: DNA Binding Domain
NAC: NAM/ATAF/CUC
NLS: Nuclear Localization Signal
NRD: Negative Regulatory Domain
SAM: Shoot Apical Meristem
TF: Transcription factor
TRR: Transcription Regulatory Region
