# Supplementary material for: The NAC side of the fruit: tuning of fruit development and maturation
Source: BMC Plant Biol. 2021 May 27;21:238. doi: 10.1186/s12870-021-03029-y (PMC8157701; doi:10.1186/s12870-021-03029-y)
Supplement: Supplementary file 1 — Additional file 1: Table S1. Number of NAC transcription factors identified in several plant species. [file 12870_2021_3029_MOESM1_ESM.docx]

**SUPPLEMENTARY INFORMATION**

**THE NAC SIDE OF THE FRUIT: TUNING OF FRUIT DEVELOPMENT AND MATURATION**

Sara Forlani, Chiara Mizzotti and Simona Masiero

Department of Biosciences, Università degli Studi di Milano, Via Celoria 26, 20133, Milan, Italy

**Table S1.** Number of NAC transcription factors identified in several plant species.

| **Species** | **Number of NACs identified** | **Reference** |
| --- | --- | --- |
| *Arabidopsis thaliana* | 100 | [1, 2] |
| *Solanum lycopersicum* | 104 | [3, 4] |
| *Solanum tuberosum* | 110 | [5] |
| *Oryza sativa* | 151 | [6] |
| *Triticum aestivum* | 359 | [7] |
| *Zea mays* | 124 | [8] |
| *Fagopyrum tataricum* | 80 | [9] |
| *Vitis vinifera* | 79 | [10] |
| *Citrus* sp. | 45 | [11] |
| *Populus trichocarpa* | 163 | [12] |
| Gossypium arboreum | 141 | [13, 14] |
| Gossypium raimondii | 145 | [15] |
| Gossypium hirsutum | 283 | [16] |
| *Gossypium barbadense* | 270 | [17] |
| *Setaria italica* | 147 | [18] |
| *Panicum virgatum* | 251 | [19] |
| *Medicago truncatula* | 97 | [20] |
| *Musa acuminata* | 162 | [21] |
| *Fragaria vesca* | 37 | [22] |
| *Manihot esculenta* | 96 | [23] |
| *Chenopodium quinoa* | 90 | [24] |
| *Cucumis melo* | 82 | [25] |
| *Citrullus lanatus* | 80 | [26] |
| *Brassica rapa* | 204 | [27] |
| *Glycine max* | 152 | [28] |
| *Nicotiana tabacum* | 152 | [29] |

**References**

1. Ooka H, Satoh K, Doi K, Nagata T, Otomo Y, Murakami K, et al. Comprehensive Analysis of NAC Family Genes in Oryza sativa and Arabidopsis thaliana. DNA Res. 2003;10:239–47.

2. Jensen MK, Kjaersgaard T, Petersen K, Skriver K. NAC genes: Time-specific regulators of hormonal signaling in Arabidopsis. Plant Signal Behav. 2010;5:907–10.

3. Kou X, Wang S, Wu M, Guo R, Xue Z, Meng N, et al. Molecular Characterization and Expression Analysis of NAC Family Transcription Factors in Tomato. Plant Mol Biol Report. 2014;32:501–16.

4. Su H, Zhang S, Yin Y, Zhu D, Han L. Genome-wide analysis of NAM-ATAF1,2-CUC2 transcription factor family in Solanum lycopersicum. J Plant Biochem Biotechnol. 2015;24:176–83. doi:10.1007/s13562-014-0255-9.

5. Singh AK, Sharma V, Pal AK, Acharya V, Ahuja PS. Genome-wide organization and expression profiling of the NAC transcription factor family in potato (solanum tuberosum L.). DNA Res. 2013;20:403–23.

6. Nuruzzaman M, Manimekalai R, Sharoni AM, Satoh K, Kondoh H, Ooka H, et al. Genome-wide analysis of NAC transcription factor family in rice. Gene. 2010;465:30–44. doi:10.1016/j.gene.2010.06.008.

7. Guérin C, Roche J, Allard V, Ravel C, Mouzeyar S, Bouzidi MF. Genome-wide analysis, expansion and expression of the NAC family under drought and heat stresses in bread wheat (T. Aestivum L.). PLoS One. 2019;14:1–26. doi:10.1371/journal.pone.0213390.

8. Fan K, Wang M, Miao Y, Ni M, Bibi N, Yuan S, et al. Molecular evolution and expansion analysis of the NAC transcription factor in zea mays. PLoS One. 2014;9:2–14.

9. Liu M, Ma Z, Sun W, Huang L, Wu Q, Tang Z, et al. Genome-wide analysis of the NAC transcription factor family in Tartary buckwheat (Fagopyrum tataricum). BMC Genomics. 2019;20:1–16.

10. Wang N, Zheng Y, Xin H, Fang L, Li S. Comprehensive analysis of NAC domain transcription factor gene family in Vitis vinifera. Plant Cell Rep. 2013;32:61–75.

11. de Oliveira TM, Cidade LC, Gesteira AS, Filho MAC, Filho WSS, Costa MGC. Analysis of the NAC transcription factor gene family in citrus reveals a novel member involved in multiple abiotic stress responses. Tree Genet Genomes. 2011;7:1123–34.

12. Hu R, Qi G, Kong Y, Kong D, Gao Q, Zhou G. Comprehensive Analysis of NAC Domain Transcription Factor Gene Family in Populus trichocarpa. BMC Plant Biol. 2010;10:1–23.

13. Shang H, Wang Z, Zou C, Zhang Z, Li W, Li J, et al. Comprehensive analysis of NAC transcription factors in diploid Gossypium: sequence conservation and expression analysis uncover their roles during fiber development. Sci China Life Sci. 2016;59:142–53. doi:10.1007/s11427-016-5001-1.

14. Fan K, Li F, Chen J, Li Z, Lin W, Cai S, et al. Asymmetric evolution and expansion of the NAC transcription factor in polyploidized cotton. Front Plant Sci. 2018;9 January:1–15.

15. Shang H, Li W, Zou C, Yuan Y. Analyses of the NAC transcription factor gene family in gossypium raimondii Ulbr.: Chromosomal location, structure, phylogeny, and expression patterns. J Integr Plant Biol. 2013;55:663–76. doi:10.1111/jipb.12085.

16. Sun H, Hu M, Li J, Chen L, Li M, Zhang S, et al. Comprehensive analysis of NAC transcription factors uncovers their roles during fiber development and stress response in cotton. BMC Plant Biol. 2018;18:1–15.

17. Liu Z, Fu M, Li H, Chen Y, Wang L, Liu R. Systematic analysis of NAC transcription factors in Gossypium barbadense uncovers their roles in response to Verticillium wilt. PeerJ. 2019;2019:e7995. doi:10.7717/peerj.7995.

18. Puranik S, Sahu PP, Mandal SN, B. VS, Parida SK, Prasad M. Comprehensive Genome-Wide Survey, Genomic Constitution and Expression Profiling of the NAC Transcription Factor Family in Foxtail Millet (Setaria italica L.). PLoS One. 2013;8:1–16.

19. Yan H, Zhang A, Ye Y, Xu B, Chen J, He X, et al. Genome-wide survey of switchgrass NACs family provides new insights into motif and structure arrangements and reveals stressrelated and tissue-specific NACs. Sci Rep. 2017;7:1–15.

20. Ling L, Song L, Wang Y, Guo C. Genome-wide analysis and expression patterns of the NAC transcription factor family in Medicago truncatula. Physiol Mol Biol Plants. 2017;23:343–56.

21. Cenci A, Guignon V, Roux N, Rouard M. Genomic analysis of NAC transcription factors in banana (Musa acuminata) and definition of NAC orthologous groups for monocots and dicots. Plant Mol Biol. 2014;85:63–80.

22. Zhang H, Kang H, Su C, Qi Y, Liu X, Pu J. Genome-wide identification and expression profile analysis of the NAC transcription factor family during abiotic and biotic stress in woodland strawberry. PLoS One. 2018;13:e0197892.

23. Hu W, Wei Y, Xia Z, Yan Y, Hou X, Zou M, et al. Genome-wide identification and expression analysis of the NAC transcription factor family in cassava. PLoS One. 2015;10:1–25.

24. Li F, Guo X, Liu J, Zhou F, Liu W, Wu J, et al. Genome-wide identification, characterization, and expression analysis of the NAC transcription factor in chenopodium quinoa. Genes (Basel). 2019;10:1–13.

25. Wei S, Gao L, Zhang Y, Zhang F, Yang X, Huang D. Genome-wide investigation of the NAC transcription factor family in melon (Cucumis melo L.) and their expression analysis under salt stress. Plant Cell Rep. 2016;35:1827–39.

26. Lv X, Lan S, Guy KM, Yang J, Zhang M, Hu Z. Global Expressions Landscape of NAC Transcription Factor Family and Their Responses to Abiotic Stresses in Citrullus lanatus. Sci Rep. 2016;6 April:1–14. doi:10.1038/srep30574.

27. Liu T, Song X, Duan W, Huang Z, Liu G, Li Y, et al. Genome-Wide Analysis and Expression Patterns of NAC Transcription Factor Family Under Different Developmental Stages and Abiotic Stresses in Chinese Cabbage. Plant Mol Biol Report. 2014;32:1041–56. doi:10.1007/s11105-014-0712-6.

28. Le DT, Nishiyama R, Watanabe Y, Mochida K, Yamaguchi-Shinozaki K, Shinozaki K, et al. Genome-wide survey and expression analysis of the plant-specific NAC transcription factor family in soybean during development and dehydration stress. DNA Res. 2011;18:263–76. doi:10.1093/dnares/dsr015.

29. Rushton PJ, Bokowiec MT, Han S, Zhang H, Brannock JF, Chen X, et al. Tobacco transcription factors: Novel insights into transcriptional regulation in the Solanaceae. Plant Physiol. 2008;147:280–95.
